# Truncated FOS impairs osteogenic differentiation and induces prostaglandin and NFκB signalling in an *in vitro* cell‐of‐origin model for osteoid osteoma and osteoblastoma

**DOI:** 10.1002/path.70010

**Published:** 2025-12-22

**Authors:** Suk Wai Lam, Tatiana Belova, Natasja Franceschini, Brendy van den Akker, David GP van IJzendoorn, Harald MM Mikkers, Hailiang Mei, Anne‐Marie Cleton‐Jansen, Marieke L Kuijjer, Karoly Szuhai, Judith VMG Bovée

**Affiliations:** ^1^ Department of Pathology Leiden University Medical Center Leiden The Netherlands; ^2^ Centre for Molecular Medicine Norway (NCMM), Nordic EMBL Partnership, University of Oslo Oslo Norway; ^3^ Department of Cell and Chemical Biology Leiden University Medical Center Leiden The Netherlands; ^4^ Department of Biomedical Data Sciences, Sequencing Analysis Support Core Leiden University Medical Center Leiden The Netherlands; ^5^ iCAN Digital Precision Cancer Medicine Flagship University of Helsinki and Helsinki University Hospital Helsinki Finland; ^6^ Department of Biochemistry and Developmental Biology University of Helsinki Helsinki Finland

**Keywords:** FOS, osteogenic differentiation, osteoid osteoma, osteoblastoma, truncated FOS, mesenchymal stem cell

## Abstract

Osteoid osteoma and osteoblastoma are non‐malignant bone‐forming tumours of the skeleton, characterised by the presence of irregular trabeculae of woven bone. Rearrangements in *FOS,* and less frequently *FOSB*, have recently been identified in osteoid osteoma and osteoblastoma. Identical rearrangements in *FO*S were previously detected in epithelioid haemangioma, where these led to truncation of the FOS protein in the C‐terminal domain, causing increased protein stability due to impaired degradation. Since FOS plays a role in osteogenic differentiation, the effect of FOS truncation on osteogenic differentiation and proliferation was investigated in an *in vitro* model for osteoid osteoma and osteoblastoma. In this model, truncated FOS (FOSΔ) was overexpressed in human foetal mesenchymal stem cells through a lentiviral vector. Osteogenic differentiation — assessed by measuring mineralisation, *ALPL* expression, and ALP activity — and proliferation rate were reduced in cells overexpressing FOSΔ compared to mesenchymal stem cells with an empty lentiviral vector (pLV). Transcriptome‐sequencing and differential gene expression analysis revealed decreased gene expression of genes in pathways involving cell cycling and mitosis and osteogenic differentiation, including WNT signalling, extracellular matrix organisation, and matrix metalloproteinases (MMPs), in FOSΔ as compared to empty vector cells, indicating decreased proliferation and osteogenesis. Instead, FOSΔ cells showed upregulation of genes involved in prostaglandin signalling and NF‐kB inflammatory pathways. These findings highlight that FOSΔ compromises cellular growth and osteogenesis, which is in line with the morphological features of osteoid osteoma and osteoblastoma with woven bone formation instead of mature lamellar bone, as well as the indolent clinical behaviour. Additionally, FOSΔ promotes inflammatory signalling instead, which correlates with clinically exquisite response to non‐steroid anti‐inflammatory drugs. © 2025 The Author(s). *The Journal of Pathology* published by John Wiley & Sons Ltd on behalf of The Pathological Society of Great Britain and Ireland.

## Introduction

Osteoid osteoma and osteoblastoma are bone‐forming tumours of the skeleton. Whereas osteoid osteomas are small in size (< 2 cm) and benign, osteoblastomas are larger and can be locally aggressive [1]. Despite differences in clinical presentation, the histology of both entities is identical. Osteoid osteoma and osteoblastoma are composed of trabeculae of osteoid and woven bone, which can show various degrees of mineralisation of the matrix [1]. These trabeculae are lined by plump osteoblast‐like cells. Between these trabeculae, highly vascularised stroma is present and usually admixed with osteoclast‐like giant cells.

**Figure 1:**
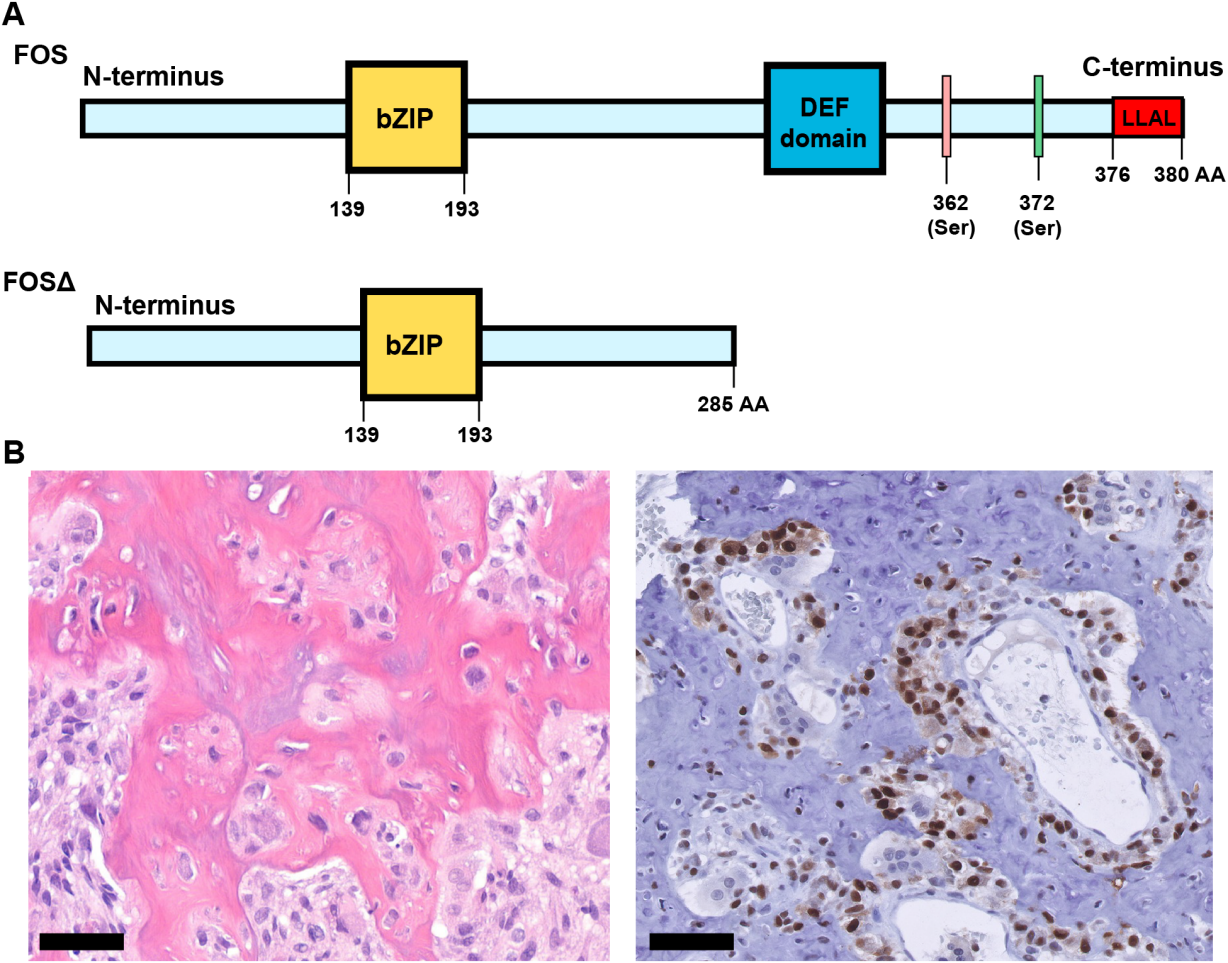
FOS is truncated and overexpressed in osteoid osteoma, osteoblastoma, and cementoblastoma. (A) Schematic overview of FOS FL protein (380 amino acids) and FOSΔ (285 amino acids) protein found in osteoid osteoma, osteoblastoma, cementoblastoma, and epithelioid haemangioma. Basic‐region Leucine Zipper (bZIP) domain, DEF domain (binding site for MAPK), and the LLAL region within the C‐terminus of FOS are indicated. The LLAL region is closely located to the phosphorylation sites Ser362 and Ser374. Figure adapted from van IJzendoorn *et al* [4] and Ofir *et al* [47]. (B) H&E shows immature woven bone deposited by osteoblasts, accompanied by osteoclasts (left panel) and FOS immunohistochemical staining (right) depicting overexpression of FOS in osteoid osteoma. Scale bars, 50 μm.

Osteoid osteoma and osteoblastoma show overexpression of FOS or FOSB due to frequent rearrangements in *FOS* (87%) and — less frequently — *FOSB* (2%) [2]. In cementoblastoma, rearrangements in *FOS* and overexpression of FOS have also been identified [3]. For osteoid osteoma and osteoblastoma, the rearrangements in *FOS* involve exon 4, leading to a truncation of the FOS protein, as stop codons are introduced near the breakpoints (Figure 1A). Rearrangements leading to the truncation of FOS at a similar position in the gene were previously identified in epithelioid haemangioma [4]. Under normal conditions, FOS is a short‐lived protein, and its levels are regulated by different post‐transcriptional and post‐translational mechanisms, including control via the 3’‐UTR region, ubiquitin‐dependent or ubiquitin‐independent proteasomal degradation, and phosphorylation [5, 6, 7, 8, 9, 10, 11].

In epithelioid haemangioma, it was shown that truncated FOS (FOSΔ) was more resistant to proteasomal degradation, caused by the absence of a highly conserved destabilising element (LLAL region) within the last four amino acids in the C‐terminus of FOS in cells expressing FOSΔ. The LLAL region acted as a signal for proteasomal degradation, causing cells carrying a mutation (FOSL376N) or deletion (FOSΔ376‐377) within the LLAL region to sustain expression of FOS protein [4, 12]. Therefore, the truncation of FOS found in osteoid osteoma, osteoblastoma, and cementoblastoma leads to stabilisation and, thus, sustained expression of FOS protein. Similar to epithelioid haemangioma, these tumours show strong nuclear expression of FOS in the tumour cells, and therefore, overexpression of FOS can be used as a diagnostic tool [13, 14] (Figure 1B).

FOS forms a heterodimer with JUN family proteins to form the AP‐1 complex and acts as a transcription factor. The AP‐1 complex is involved in cell growth control and cell transformation and a plethora of other essential cellular processes [15, 16, 17]. Sustained FOS expression is sufficient for the transformation of cells [18]. Interestingly, transgenic mice overexpressing FOS in bone develop osteosarcoma, although FOS overexpression itself is rarely identified in human osteosarcoma [13, 14, 19, 20, 21]. The AP‐1 complex and FOS also play a role in osteogenic differentiation. During osteogenic differentiation, the expression of FOS increases, which in turn further affects the expression of other genes involved in osteogenic differentiation [22, 23]. For example, the promoters of key osteogenic genes such as alkaline phosphatase, collagen I, and osteocalcin all have an AP‐1 site [23]. AP‐1 was previously identified as a driver of tumorigenesis in vascular tumours, epithelioid haemangioma, and pseudomyogenic haemangioendothelioma [4]. It was found that human umbilical vein endothelial cells (HUVECs) expressing FOSΔ displayed increased endothelial sprouting without changing proliferation. As vascular tumours harbour similar *FOS*, or *FOSB* translocations, we hypothesise that truncation of FOS in osteoid osteoma and osteoblastoma might drive tumorigenesis by influencing osteogenesis, leading to the characteristic formation of immature woven bone. To test our hypothesis, we studied how alterations in FOS affect osteogenic differentiation and/or maturation and possibly proliferation by creating a cell‐of‐origin‐based model overexpressing FOS.

## Materials and methods

### Ethics approval

Foetal mesenchymal stem cells (fMSCs) were derived from the femurs of a 22‐week‐old deceased foetus (abortion with unknown medical cause) and were collected based on individual written parental informed consent after approval by the Medical Ethics Committee of the Leiden University Medical Center (reference number P08‐087). The experiments involving human materials were performed in accordance with the principles outlined in the Declaration of Helsinki [24].

### Cell culture

Since MSCs are the most likely progenitor cells of osteogenic tumours [25], fMSCs were cultured to overexpress FOSΔ. The cells were isolated following a protocol similar to the adult MSC isolation method previously described [26]. In brief, the femoral shafts were flushed with HEPES‐buffered saline solution, and the flushed cell suspension was filtered through a mesh filter. Cells were centrifuged for 5 min at 1,000 rpm and seeded in complete αMEM (Gibco, Grand Island, NY, USA) medium on 15‐cm culture dishes. Once subconfluent, cells were passaged at a 1:3 ratio using trypsin. fMSCs were validated by flow cytometry as positive for MSC markers CD44, CD90, and CD105 and negative for haematopoietic marker CD45. fMSCs (passage number 5–13) were cultured in αMEM (Gibco) supplemented with 10% FBS, 1% non‐essential amino acids (Gibco), and 1% pen/strep (Gibco) in a humidified incubator at 37 °C with 5% CO_2_. Cells were tested for mycoplasma contamination.

### Lentiviral transduction

FOS constructs were generated as previously described [4]. In brief, four human FOS cDNAs were used, containing full‐length FOS (FOS FL), a FOS isoform lacking the C‐terminal 95 amino acids but including the bZIP domain (FOSΔ, Figure 1A), a FOS isoform with a mutation (FOSL376N), or a FOS isoform with a deletion (FOSΔ376‐377) within the C‐terminal four amino acids (LLAL) containing a helical region. These cDNAs were C‐terminally fused in frame with a FLAG tag and cloned into a cytomegalovirus (CMV)‐driven pLV using BstBI and NheI restriction sites. Lentivirus was produced in HEK 293 T cells using a PEI‐based co‐transfection protocol and harvested at 48 and 72 h after transfection. Viral supernatants were filtered and quantified by p24 ELISA (BioRad, Hercules, CA, USA) at the Leiden University Medical Center, and equal amounts of viral particles were used to transduce fMSCs at an intended multiplicity of infection (MOI) of 5, ensuring comparable delivery across constructs. Transduction was performed in the presence of polybrene (8 μg/ml) (Sigma‐Aldrich, Burlington, MA, USA), and the following day, the medium was replaced and cells were selected with puromycin (5 ng/ml) (Thermo Fisher Scientific, Waltham, MA, USA). Untransduced fMSCs and fMSCs transduced with pLV served as controls.

### Osteogenic differentiation

Cells were seeded at 5,000 cells/cm^2^ for osteogenic differentiation. One day after seeding, cells were treated with osteogenic compounds: β‐glycerophosphate (5 mm, Sigma‐Aldrich), dexamethasone (0.1 μm, Sigma‐Aldrich), and ascorbate‐2‐phosphate (0.15 mm, Sigma‐Aldrich). Medium with osteogenic compounds was refreshed twice a week. As a negative control, cells were cultured without osteogenic compounds. Osteogenic differentiation experiments were performed independently at least three times by three independent researchers.

### Alkaline phosphatase activity assay

After 10 days of osteogenic differentiation, cells were lysed with PBS/Triton 0.1% and incubated with pNPP (P7998, Sigma‐Aldrich) for 4 min. The reaction was stopped with NaOH, and absorption at 405 nm was measured using a microplate reader (Infinite M Plex, Tecan Group Ltd., Zürich, Switzerland). Experiments were performed in triplicate and repeated independently at least three times.

### Alizarin Red staining

After 3 weeks of osteogenic differentiation, mineralisation was assessed by Alizarin Red S staining (Sigma‐Aldrich). Cells were fixed in cold ethanol and stained (2 g Alizarin Red S in 100 ml water, pH 4.2) for 5 min. For quantification, staining was extracted with 10% acetic acid, heated (85 °C, 10 min), centrifuged (12,000 rpm, 15 min), and neutralised with 10% ammonium hydroxide. Absorbance was measured at 405 nm (Infinite M Plex, Tecan). Experiments were performed in triplicate and repeated independently at least three times.

### Cell proliferation assays

For proliferation assays, 1,500 cells/well were seeded in 96‐well plates. At days 1–5, cells were incubated with PrestoBlue (Invitrogen, Waltham, MA, USA) for 60 min, and fluorescence was measured (Infinite M Plex, Tecan). Cells were then fixed using 4% formaldehyde, stained with Hoechst (2 μg/ml), and nuclei were quantified using the Cellomics ArrayScan VTI HCS 700 (Thermo Fisher Scientific, Waltham, MA, USA). Experiments were performed in triplicate and repeated independently at least three times.

### RT‐qPCR

RNA was extracted at weeks 0, 1, 2, and 3 of osteogenic differentiation using TRIzol (Invitrogen, Catalogue No. 15596026) following the manufacturer's protocol. cDNA was synthesised with the iScript cDNA Synthesis Kit (Bio‐Rad, 1708890) following the manufacturer's protocol. RT‐qPCR was performed using iQ SYBR Green Supermix (Bio‐Rad, 1708880) on a Bio‐Rad thermal cycler with primers for *FOS*, *ALPL*, and *GAPDH* (reference gene). Primer sequences are provided in the supplementary material, Table S1. Relative gene expression levels to reference genes were determined with the following formula: 2^(Ct value reference gene − Ct value gene of interest)^. Experiments were performed in duplicate and repeated independently at least twice.

### Histochemistry and immunohistochemistry

Histological analysis followed standard protocols. Formalin‐fixed paraffin‐embedded sections were stained with haematoxylin and eosin (H&E) (Tissue‐Tek Prisma® Plus Automated Slide Stainer, Sakura, Alphen aan den Rijn, Zuid‐Holland, The Netherlands). Additionally, sections were subjected to FOS immunohistochemistry using a rabbit polyclonal anti‐FOS antibody (clone F7799, 1:6,000, Merck, Burlington, MA, USA) with Tris‐EDTA (pH 9.0) antigen retrieval, followed by DAB detection and haematoxylin counterstaining.

### RNA‐sequencing and data analysis

Total RNA‐sequencing using paired‐end reads was performed by GenomeScan (Leiden, The Netherlands) on the NovaSeq6000 platform (Illumina, San Diego, CA, USA) for FOS FL, FOSΔ, a FOS isoform with an L376N mutation (FOS mut), untransduced fMSC (fMSC), and fMSC with pLV at weeks 0, 1, 2, and 3 in triplicate (total of 60 samples). Raw RNA‐seq data were processed using the RNA‐seq BioWDL pipeline version 5.0.0 developed by the SASC team at LUMC (https://biowdl.github.io/, LUMC, Leiden, The Netherlands). Reads were aligned against GRCh38 with STAR (version 2.7.5a). UMI‐based duplicate removal was performed using UMI‐tools (version 1.1.1). Expression quantification was performed using HTseq‐count (version 0.12.4) with Ensembl gene annotation version 105 [27]. Gene counts were normalised with the ‘median of ratios’ method in the DESeq2 package version 1.36.0 in R [28]. Normalised expression values were log2‐transformed and averaged across all biological replicates.

To study the individual contribution of endogenous *FOS* and exogenous *FOS*, for each FOS FL, FOSΔ, pLV, and fMSC sample, all alignment reads overlapping with the start position of the open reading frame of the first coding exon of *FOS* gene (GRCh38 chr14:75278983) were extracted. A customised Python script was implemented to categorise these alignment reads based on the CIGAR string and calculate the number of alignment reads for two different categories named softclipped (exogenous *FOS*) and fully matched (exogeneous *FOS*). In this analysis, a paired alignment and a singleton alignment were both counted only once. After excluding spliced reads, softclipped reads contained at least six softclipped bases, indicating the presence of reads containing the artificial FLAG sequence tag (GACTACAAGGATGACGACGATAAG). Fully matched reads fully aligned to the 5’‐UTR of *FOS* exon 1 reference genome, indicating that these reads did not contain the artificial sequence tag.

Multidimensional scaling (MDS) plots were generated to visualise sample clustering based on gene expression profiles for all samples, including replicates, as well as the average gene expression across replicates. Differential gene expression analysis was performed using the edgeR package version 3.40.2 in R, identifying genes as differentially expressed (DEGs) with absolute log2Fold Change ≥ 1 and FDR < 0.01 [29]. Differential gene expression analysis was performed by comparing wild‐type fMSCs, pLV, FOS FL, FOS mut, and FOSΔ at different time points.

Functional enrichment analysis of DEGs for biological processes or Kyoto Encyclopedia of Genes and Genomes (KEGG) pathways [30] was conducted using the GOstats package version 2.62.0 in R [31]. The significance of each biological process or pathway was assessed through a hypergeometric test, implemented via the ‘*GOHyperGParams*’ function within the GOstats package version 2.62.0. *p* values were adjusted for multiple testing using the Benjamini–Hochberg method. All biological processes and pathways with FDR‐adjusted *p* values <  0.01 were considered significantly enriched. Gene set enrichment analysis was performed on ranked gene lists from edgeR using the gfsea package version 1.24.0 in R, with human hallmark gene sets (REACTOME pathways version 7.1) obtained from the MSigDB database. For data visualisation R packages ggplot2 (version 3.5.1), EnhancedVolcano (version 1.16.0), and pheatmap (version 1.0.12) were used [32]. All software programs were last accessed on 15 September 2025. The RNAseq dataset of all 60 samples is deposited at EGA (https://ega-archive.org/datasets/EGAD50000001839).

### Statistical analyses

Statistical analyses for cell culture experiments were performed using GraphPad Prism version 9 (GraphPad Software, San Diego, CA, USA). For multiple comparisons between groups, the Kruskal–Wallis test was applied, with all groups compared to cells transduced with pLV. A *p* value < 0.05 was considered statistically significant.

## Results

### Mesenchymal stem cells expressing FOSΔ have reduced osteogenic differentiation capacity

**Figure 2:**
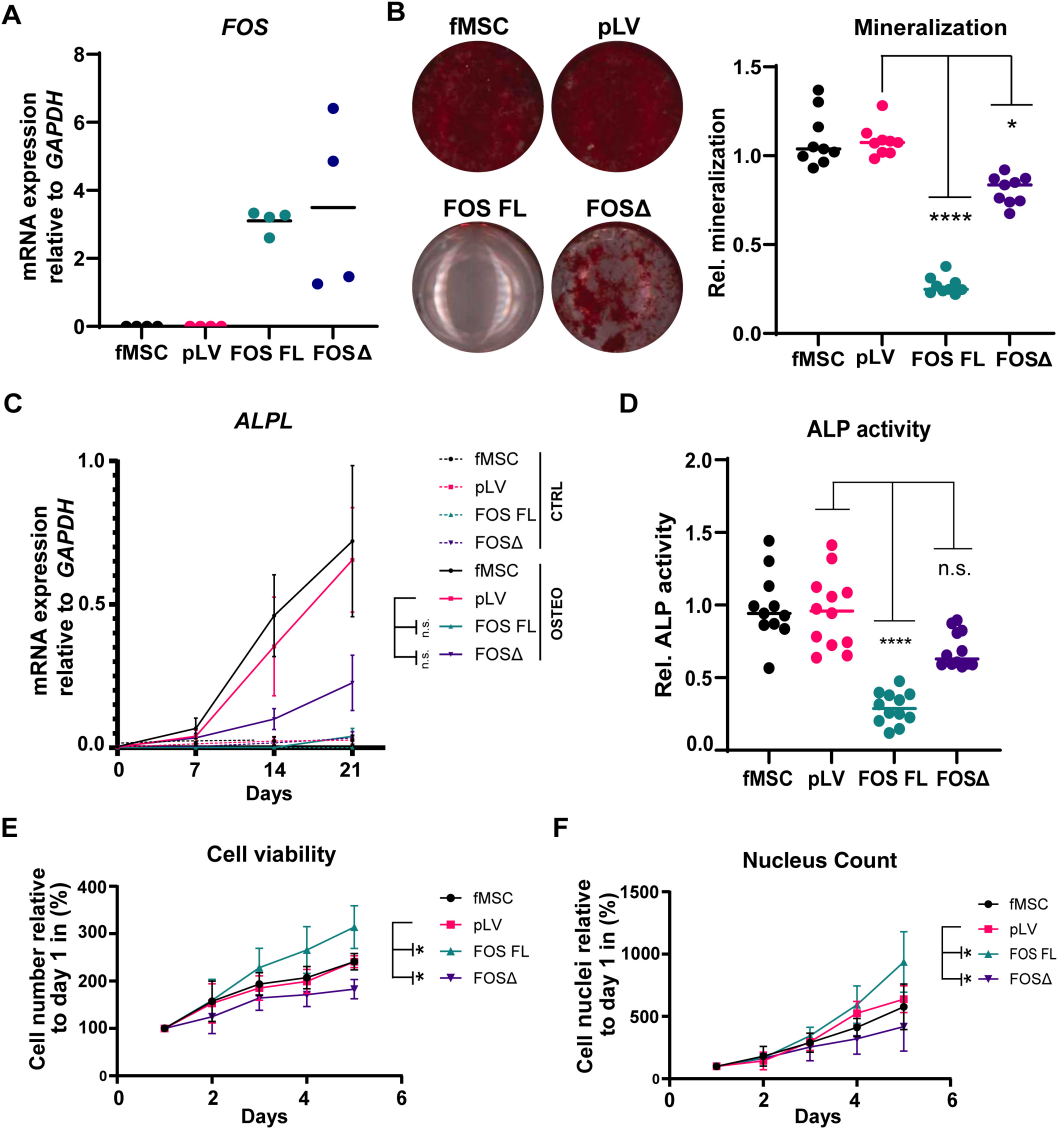
Osteogenic differentiation is reduced upon overexpression of FOSΔ. (A) *FOS* mRNA was overexpressed in fMSCs transduced with FOS FL or FOSΔ. (B) fMSCs expressing FOSΔ show reduced mineralisation, while mineralisation is almost completely absent in fMSCs expressing FOS FL. Mineralisation was quantified relative to pLV fMSCs. (C) Expression of osteogenic marker *ALPL* is induced during osteogenic differentiation. (D) Alkaline phosphatase activity was determined in fMSCs relative to pLV. (E,F) The cell viability and nucleus count of fMSCs with various constructs over time. Both were reduced in fMSCs expressing FOSΔ compared to untransduced (fMSC) and empty vector transduced (pLV) fMSCs, whereas this was increased in cells expressing FOS FL. Each dot represents a single data point and a line represents the mean ± SD. Multiple independent experiments were performed in triplicate. Groups were compared to pLV using Kruskal–Wallis test. *p ≤ 0.05; ****p ≤ 0.0001; n.s., not statistically significant, *p* > 0.05.

fMSCs transduced with constructs containing FOS FL or FOSΔ showed overexpression of *FOS* at the mRNA level (Figure 2A) and strong nuclear staining by immunohistochemistry (supplementary material, Figure S1). As expected, no significant differences were observed in expression levels between the two constructs, reflecting their comparable design driven by the constitutive CMV promoter within the lentiviral vector system. The high expression observed in these samples reflects exogenous expression driven by the constructs. In contrast, FOS expression in the control groups (untransduced fMSCs and pLV) remained very low. This low expression aligned with the endogenous regulation of FOS, an immediate early gene typically expressed transiently and at low levels under basal conditions (supplementary material, Table S2). These cells were then used to determine the effect of FOS truncation on osteogenic differentiation. After 3 weeks of culture in osteogenic medium, Alizarin Red staining revealed that fMSCs expressing FOSΔ showed a reduction of mineralisation (*p* = 0.02), whereas fMSCs expressing FOS FL lost osteogenic differentiation capacity, almost completely lacking mineralisation (*p* ≤ 0.0001) (Figure 2B). *ALPL* RNA expression and ALP activity showed a trend towards reduction in fMSCs overexpressing FOSΔ compared to pLV, although this was not statistically significant (*p* = 0.2 for *ALPL* expression and *p* = 0.1 for ALP activity) (Figure 2C,D). fMSCs overexpressing FOS FL further reduced *ALPL* expression (although not significant, *p* = 0.2) and ALP activity (*p* ≤ 0.0001) compared to wild‐type fMSCs (pLV) (Figure 2C,D).

### Mesenchymal stem cells expressing FOSΔ show decreased proliferation

Since sustained expression of FOS is known to induce transformation of cells [18, 33], the proliferation rate of fMSCs expressing FOS FL or FOSΔ was determined. Cell viability, as well as nucleus count, was assessed over a period of 5 days. FOSΔ showed lower proliferation rates compared to wild‐type fMSCs (*p* ≤ 0.05), whereas fMSCs expressing FOS FL showed an increase in proliferation rate (*p* ≤ 0.05) (Figure 2E,F).

### Mutations or deletions in the C‐terminal region of FOS protein impair osteogenic differentiation capacity similar to FOSΔ

**Figure 3:**
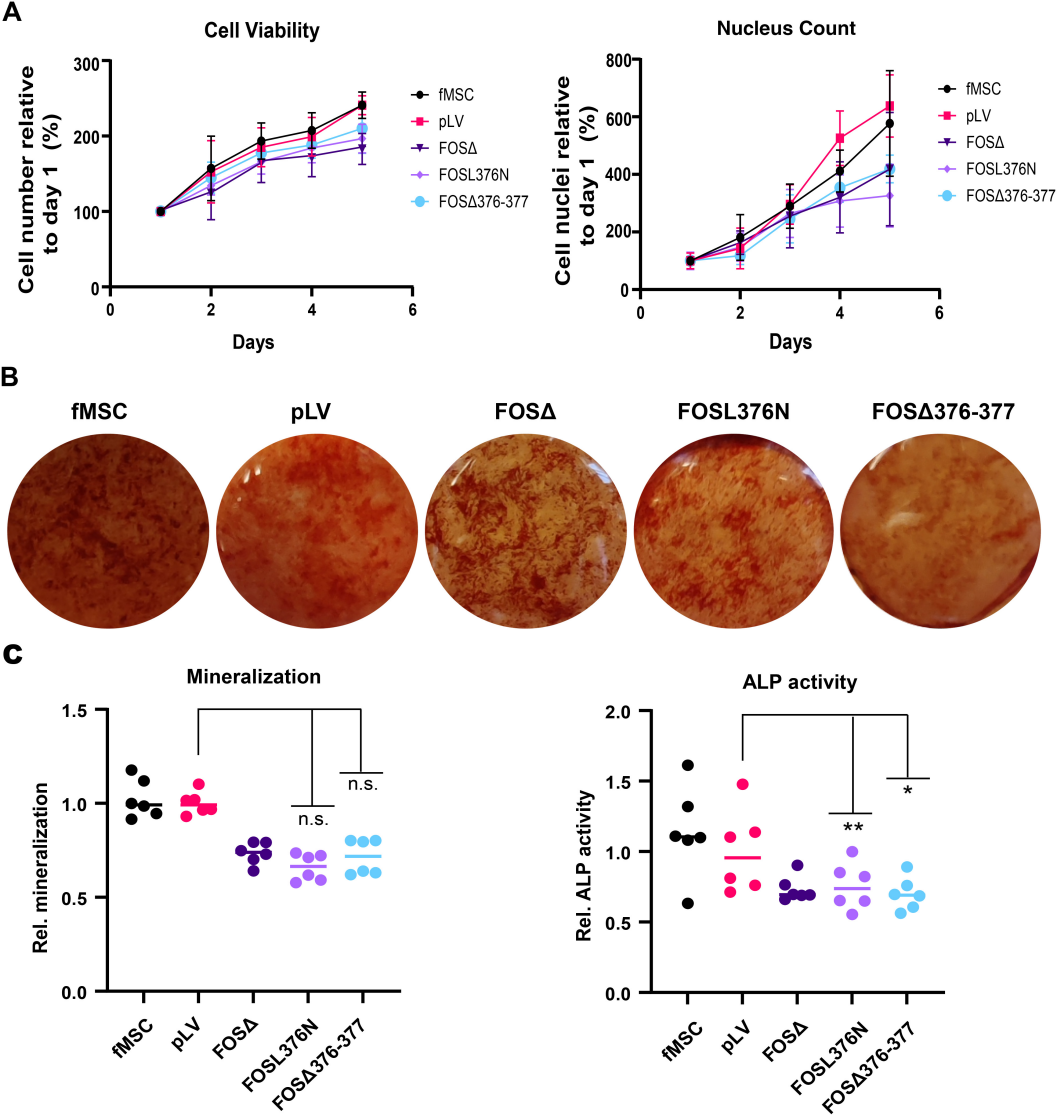
Osteogenic differentiation capacity and proliferation of fMSCs expressing FOS harbouring alterations in LLAL region are comparable with fMSC overexpression of FOSΔ. (A) Cell viability and nucleus count showed a similar proliferation rate in fMSCs expressing FOSΔ, FOS with a deletion (FOSΔ376‐377), or FOS with a mutation (FOSL376N) in the LLAL region of C‐terminal domain. (B) Mineralisation, as measured by Alizarin Red staining of FOSL376N or FOSΔ376‐377, was similar to FOSΔ. Mineralisation was quantified relative to pLV transduced fMSCs. (C) Alkaline phosphatase activity was determined in fMSCs, relative to empty vector transduced fMSCs (pLV), which was comparable among fMSCs expressing FOSΔ or FOS with a deletion (FOSΔ376‐377) or mutation (FOSL376N) in the LLAL region of C‐terminal domain. Each dot represents a single data point and a line represents the mean ± SD. Multiple independent experiments were performed in triplicate. Groups were compared to pLV using Kruskal–Wallis test. *p ≤ 0.05; **p ≤ 0.01; n.s., not statistically significant, *p* > 0.05.

Proliferation rate and osteogenic differentiation capacity were determined for additional FOS constructs to explore whether disruption of the C‐terminal region of FOS protein could explain the changes in proliferation and osteogenic differentiation in fMSCs expressing FOSΔ. fMSCs expressing FOSL376N or FOSΔ376‐377, which are both alterations located within the LLAL region of the C‐terminus of FOS, showed a similarly diminished proliferation rate (Figure 3A) and osteogenic differentiation capacity as fMSCs expressing FOSΔ, as measured by mineralisation content (Figure 3B) and alkaline phosphatase activity (Figure 3C). These results indicate that disruption of the helical region within the C‐terminus of FOS, leading to an increased stability, is responsible for the impairment of osteogenic differentiation capacity.

### Identification of DEGs

**Figure 4:**
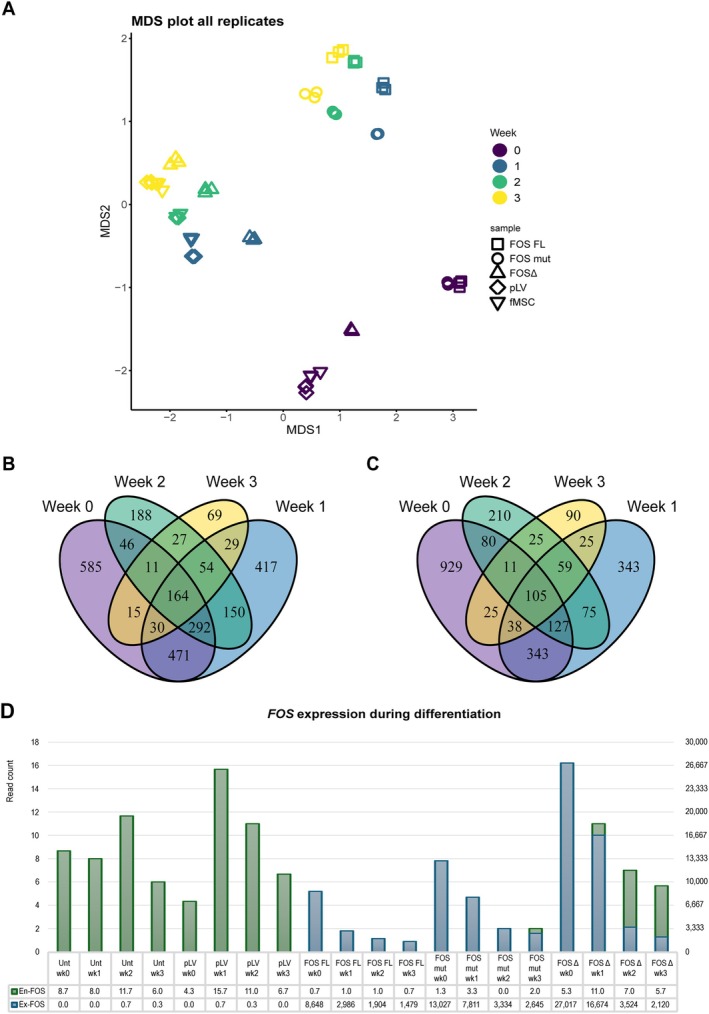
Clustering patterns and differential gene expression of FOS constructs across differentiation weeks. (A) MDS plots based on gene expression profiles illustrate tight clustering of replicates for each construct across different time points. Notably, samples used as controls (fMSC and pLV) also exhibited tight clustering. MDS analysis indicates that overall gene expression profiles of FOS FL and mutant FOS (FOS mut) (L376N) were more similar to each other than to FOSΔ. (B,C) Venn diagrams illustrate number of (B) upregulated DEGs and (C) downregulated DEGs in FOSΔ compared to pLV across differentiation weeks, with distinct colours representing each time point (week 0 in purple, week 1 in blue, week 2 in green, and week 3 in yellow). Non‐overlapping sections indicate count of unique genes specific to each week, while overlapping areas display number of shared DEGs across two or more weeks. (D) High expression of *FOS* observed in FOS FL, FOS mut and FOSΔ samples reflecting exogenous expression driven by the constructs. In contrast, *FOS* expression in the controls remained very low. Additionally, in all samples *FOS* expression is increased during the early stages of osteogenic differentiation and subsequently decreased during the mineralisation phase.

To explore the effect of the truncation of *FOS* on the transcriptome level, RNA‐sequencing was performed on fMSCs transduced with FOSΔ, FOS FL, FOS mut (L376N), empty vector (pLV), and untransduced controls (fMSC), collected at weeks 0, 1, 2, and 3 of osteogenic differentiation, in triplicate. The log2‐transformed normalised expression values were averaged over all biological replicates. MDS plots based on gene expression profiles show tight clustering of the replicates for each construct for the different time points. Tight clustering was observed between pLV and untransduced controls. FOS FL and FOS mut were more similar to each other than to FOSΔ (Figure 4A). Paired differential gene expression analysis between FOSΔ and pLV already revealed 3,272 DEGs prior to differentiation (week 0), and 1,658 genes were downregulated, while 1,614 were upregulated in FOSΔ. During differentiation, the amount of DEGs declined from 1,115 downregulated genes (week 1) to 692 genes (week 2) and 378 genes (week 3). Similarly, 1,607 genes, 932 genes, and 399 genes were upregulated in FOSΔ during week 1 to 3 respectively (Figure 4B,C). The amount of unique DEGs and shared DEGs between the two constructs declined upon differentiation.

### Osteogenic pathways are downregulated in FOSΔ, while the prostaglandin and NFκB pathways are upregulated

**Figure 5:**
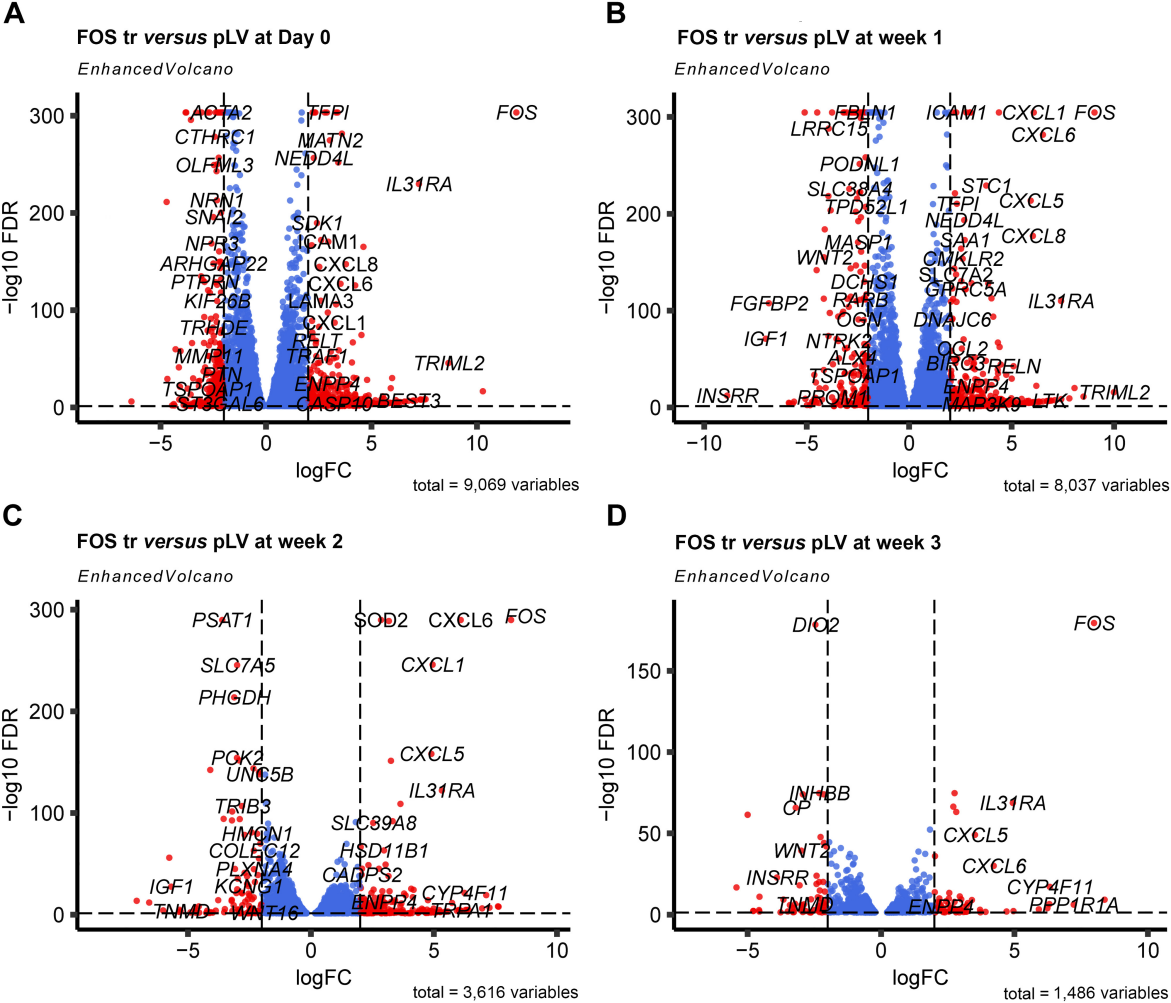
Volcano plot of DEGs identified between FOSΔ and pLV at (A) week 0, (B) week 1, (C) week 2, and (D) week 3.

**Figure 6:**
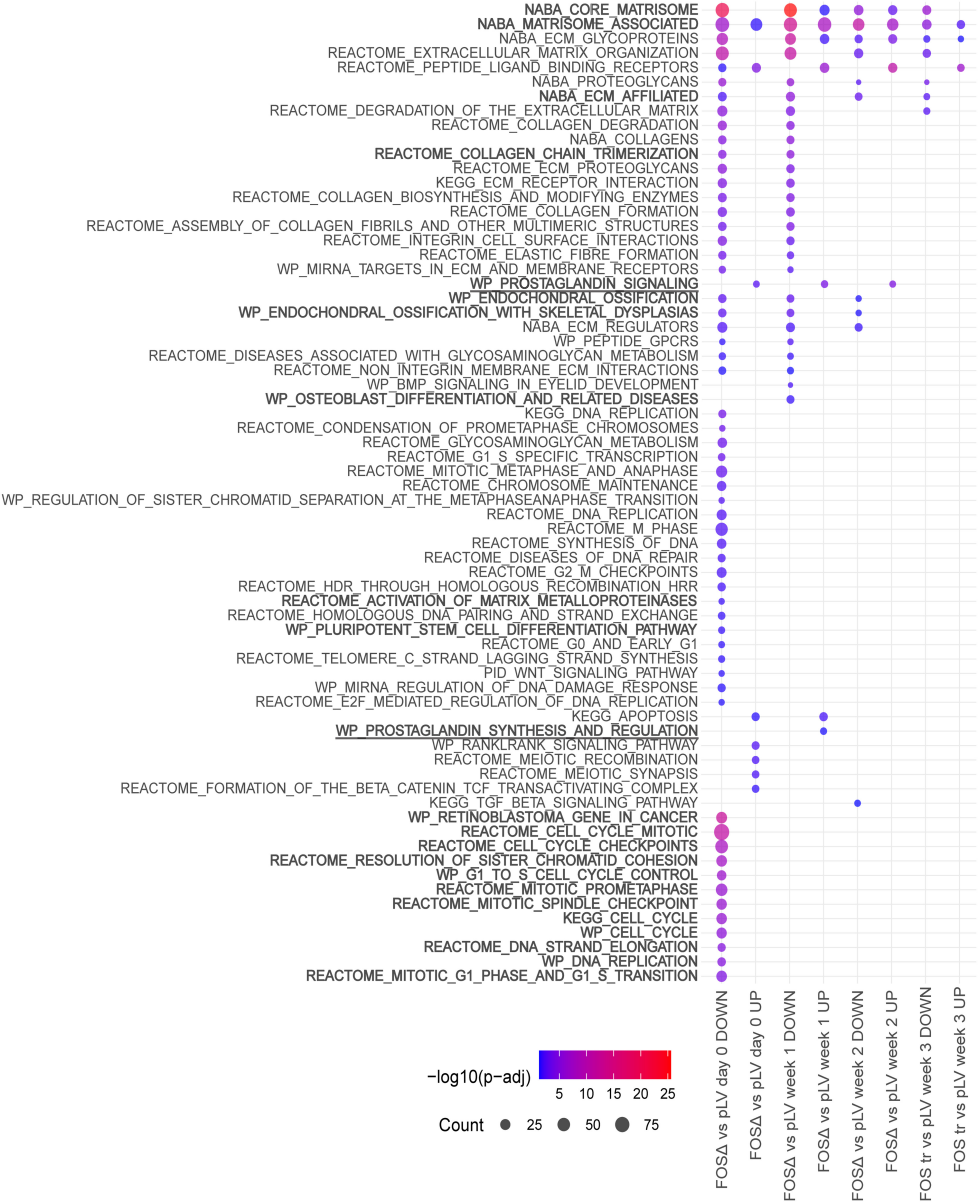
Selection of enriched pathways illustrated in bubble plot in which −log10 adjusted *p* values are visualised using a colour scale and gene count in bubble size. FOSΔ shows multiple downregulated pathways involving proliferation and osteogenesis compared to pLV, while the prostaglandin signalling pathway is notably enriched in FOSΔ.

Further analysis of the top 100 DEGs revealed consistent upregulation of *FOS* in FOSΔ compared to pLV across all time points, as expected. Consistent with previous findings, in all constructs the *FOS* expression increased during the early stages of osteogenic differentiation and subsequently decreased during the mineralisation phase (supplementary material, Table S2; Figure 4D). Prior to differentiation at week 0, genes associated with promoting cell growth and division, such as *PIMREG*, *PTPRN*, and *GLI1*, were downregulated in FOSΔ compared to pLV, consistent with the lower proliferation rates in FOSΔ relative to pLV (Figure 5; supplementary material, Data S1 and Data S2). Enrichment analysis of biological processes using all DEGs further supported this trend, as pathway analysis using various gene sets (Gene Ontology, KEGG, WikiPathways, NABA, and Reactome) (https://geneontology.org, https://wikipathways.org, [30, 34], https://reactome.org) showed significant depletion of pathways related to cell cycling, mitosis, and DNA repair in FOSΔ compared to pLV. Additionally, pathways related to collagen synthesis and degradation, matrix metalloproteinases, extracellular matrix (ECM) organisation, and WNT signalling were also less enriched in FOSΔ (Figure 6; supplementary material, Data S3).

Upon osteogenic differentiation, these pathways remained downregulated in FOSΔ, in addition to pathways associated with collagen chain trimerisation, osteoblast differentiation, ossification, and ECM organisation. Genes relevant to osteogenesis, such as *ASPN*, *ALX4*, *PRKG2*, *TMEM119*, *RANBP3L*, and *WNT2*, were also underexpressed in FOSΔ relative to pLV. This pattern persisted throughout the second and third weeks of differentiation, with the downregulation of additional WNT pathway genes such as *DKK2*, *WNT2*, and *WNT16* in FOSΔ, further confirming the impaired osteogenic potential (Figures 5 and 6; supplementary material, Data S1). Interestingly, pathways related to ECM and collagen degradation were enriched in pLV throughout differentiation, potentially reflecting a counterbalance mechanism as part of bone remodelling (Figure 6; supplementary material, Data S3).

In line with these findings, FOSΔ also exhibited distinct expression dynamics in genes related to proliferation and osteoclast regulation. Among the top 100 DEGs, genes with antiproliferative properties, such as *BTG4*, were upregulated in FOSΔ prior to differentiation and remained highly expressed in week 1, suggesting suppression of cell proliferation. Interestingly, after 2 weeks of differentiation, *TNFRSF11A*, a key mediator of osteoclast development, was upregulated in FOSΔ, while by week 3, *PRXL2A*, an inhibitor of osteoclast differentiation, also showed increased expression, indicating complex regulation of osteoclast activity in FOSΔ (supplementary material, Data S1).

Remarkably, among the few upregulated pathways in FOSΔ, the pathway involving prostaglandin synthesis and regulation was enriched in FOSΔ during differentiation (adjusted *p* = 0.04 for week 0, adjusted *p* = 0.00009 for week 1, and adjusted *p* = 0.00004 for week 2). In line with this finding, pathways involving pro‐inflammatory and profibrotic mediators were also enhanced (Figure 6; supplementary material, Data S3). After further analysis of the genes involved in the prostaglandin synthesis pathway, different pro‐inflammatory genes of the NFκB signalling pathway (*NLRP3*, *NFKB1*), members of the CXC chemokine family (i.e. *CXCL1*, *CXCL8*, *CXCL9*, *CXCL10*), members of the interleukin family (i.e. *IL1A, IL1B, IL6, IL12A, IL17F*), genes involved in prostaglandin synthesis (*PTGES*), monocyte chemotaxis (*CCR2*), and regulation (*CSF1*) were upregulated. Notably, *VEGFA*, which is involved in angiogenesis, mM*P9*, responsible for the degradation of the extracellular matrix, and *TGFB1*, which regulates cellular proliferation and differentiation, were found to be downregulated in FOSΔ (supplementary material, Figure S2).

Pathway analysis comparing FOS FL and pLV revealed significant downregulation of osteoblast differentiation pathways in FOS FL, while in pLV these pathways were enriched together with both up‐ and downregulation of genes involved in matrix organisation and degradation. When comparing FOSΔ to FOS FL, FOSΔ showed a reduced enrichment of pathways related to cell cycling and mitosis at week 0 and increased enrichment in pathways associated with osteogenic differentiation (supplementary material, Data S4). These results are in line with the previous cell culture experiments. As mutations in the LLAL region and the overexpression of FOS FL have not been described in osteoid osteoma or osteoblastoma, further data analysis was not conducted to explore these specific conditions.

## Discussion

Osteogenesis is a complex process involving multiple pathways and effectors, including FOS. The changes in the expression level of FOS over the course of bone formation are important in normal osteogenesis, as the expression levels of FOS are variable during the different stages of bone formation. While a high expression of FOS is important during proliferation, the expression decreases rapidly during matrix maturation and is barely present during mineralisation [35]. This dynamic regulation of FOS is essential for normal osteogenesis, as evidenced by findings in FOSΔ, where sustained expression led to downregulation of key osteogenic pathways like BMP and WNT and, ultimately, impaired bone formation. These results underscore the importance of fluctuating FOS expression in normal osteogenesis and support a causative link between the FOS translocation and the characteristic presence of immature woven bone in osteoid osteoma and osteoblastoma.

Another interesting finding is the enrichment of the pathway involving prostaglandin signalling, which was upregulated among the few enriched pathways in FOSΔ. We found a high expression of prostaglandin E synthase (*PTGES*), which codes for an enzyme converting prostaglandin H2 to prostaglandin E [36]. Osteoblastoma, but especially osteoid osteoma, is characterised by severe nocturnal pain that is exquisitely responsive to non‐steroid anti‐inflammatory drugs (NSAIDs). These drugs exert their function by inhibiting the synthesis of prostaglandin, a lipid mediator which plays a role in the generation of inflammatory and neuropathic pain. In line with current results, previous studies also showed higher levels of prostaglandin E2, prostaglandin F2, and prostacyclin in the nidus of osteoid osteoma compared to normal bone [37, 38], which may explain the remarkable sensitivity to NSAIDs. The current study suggests that truncation of FOS due to translocation is driving the upregulation of prostaglandins in these tumours.

It is known that AP‐1, together with NFκB and others, generally acts as an activator of pro‐inflammatory genes, regulating the attraction and activation of immune cells. The AP‐1 transcription factor connects inflammatory processes to the activation of signalling pathways in osteoclasts, as the differentiation of osteoclasts depends on several cytokines, such as M‐CSF, TNF‐α, interferons, and interleukins [39, 40]. In line with this, these effectors of the pro‐inflammatory NFκB signalling pathway were enriched in FOSΔ throughout differentiation. Interestingly, when correlating this to the morphological features of osteoid osteoma and osteoblastoma, osteoclast‐like giant cells are nearly always conspicuously present in these tumours [41].

Additionally, in epithelioid haemangioma, which harbours *FOS* fusions, these ‘bystander’ cells are observed, as the presence of eosinophils is a key morphological feature of this vascular tumour [4, 42]. The activation of c‐FOS, c‐JUN, and NFκB through the interaction of IL‐5, a critical mediator of eosinophilic inflammation, with the IL‐5 receptor (IL‐5R), promotes eosinophil survival, proliferation, and differentiation [43, 44]. Consequently, the presence of eosinophils in epithelioid haemangioma is likely driven by the sustained overexpression of FOS. The absence of eosinophils in osteoid osteoma and osteoblastoma further underscores the complex and multifaceted role of AP‐1 in immune regulation.

Other clinical features of osteoid osteoma and osteoblastoma include slow growth and non‐malignant behaviour. In line with this, fMSCs overexpressing FOSΔ showed reduced proliferation, as well as downregulation of pathways involving proliferation and cell cycling. The difference in osteogenic differentiation and proliferation as a result of overexpressing FOS FL or FOSΔ in fMSCs is interesting. It is known that sustained expression of FOS FL leads to the transformation of cells [18, 33]. fMSCs overexpressing FOS FL showed increased proliferation compared to FOSΔ and wild‐type cells, which was also reported in a previous study using MSCs [45]. As differentiation and proliferation are carefully balanced cellular processes [46], it is not surprising that the same fast‐growing cells lose osteogenic differentiation capacity. The difference in proliferation and differentiation observed in fMSCs expressing truncated or FOS FL fits the phenotypical difference between osteosarcoma, a malignant bone tumour, and the non‐malignant bone‐forming tumours osteoid osteoma and osteoblastoma [1]. These data suggest that the definitive phenotype is a careful balance between differentiation and proliferation.

Of note, not only fMSCs expressing FOSΔ but also fMSCs expressing FOS with a disrupted helical region of the C‐terminus (FOSL376N and FOSΔ376‐377) exhibited similar phenotypical results, characterised by impaired osteogenesis and a reduced proliferation rate. As the helical region of FOS contains a signal for proteasomal degradation, these results suggest that the disruption of the helical region impairs osteogenic differentiation. However, at the transcriptional level, mutant FOS appears to behave more similarly to FOS FL. This suggests an important, however not yet elucidated, function of the DEF domain in the C‐terminus of FOS, which is lacking in FOSΔ, warranting further research. Notably, to date, mutations in the helical region of the C‐terminus have not been identified in translocation‐negative osteoid osteoma and osteoblastoma [2].

To conclude, truncation of FOS in fMSCs results in a lower proliferation rate and impaired osteogenic capacity as evidenced on the cellular and transcriptional level. In addition, upregulation of the prostaglandin and NFκB pathways were found when FOS is truncated. These results reflect the clinical and morphological hallmarks of osteoid osteoma and osteoblastoma, which are slow‐growing tumours with exquisite responses to NSAIDs and characterised by the presence of immature bone; thus, they strongly support the driving role of FOS in tumorigenesis in osteoid osteoma and osteoblastoma.

## Author contributions statement

SWL, NF, JVMGB, KS, A‐MC‐J and DGPvI contributed to the conceptualisation of the study. SWL, NF, TB, HMMM, BA, MLK and HM performed experiments and data analysis. SWL, NF, JVMGB, KS and A‐MC‐J prepared the original draft of the manuscript. SWL, NF, BA, DGPvI, JVMGB, KS, A‐MC‐J, HMMM, TB, MLK and HM reviewed, edited and approved the final version of the manuscript.

## Supporting information


**Figure S1.** Histology and FOS immunohistochemistry of fMSCs
**Figure S2**. Heatmap for genes relevant in prostaglandin synthesis pathway, in which clear upregulation of different genes is present in FOSΔ compared to pLV
**Table S1**. Primers

**Table S2.** Endogenous and exogenous *FOS* expression (provided as separate Excel file)

**Data S1.** Top 100 DEGs in FOSΔ *versus* pLV (provided as a separate Excel file)

**Data S2.** Complete list of DEGs in FOSΔ *versus* pLV (provided as a separate Excel file)

**Data S3.** Enriched pathways in FOSΔ *versus* pLV (provided as a separate Excel file)

**Data S4.** DEGs and enriched pathways in FOSΔ *versus* FOS FL and pLV *versus* FOS FL (provided as a separate Excel file)

## Data Availability

The RNAseq dataset of all 60 samples is deposited at EGA (https://ega-archive.org/datasets/EGAD50000001839).
