# Supplementary material for: Truncated FOS impairs osteogenic differentiation and induces prostaglandin and NFκB signalling in an in vitro cell‐of‐origin model for osteoid osteoma and osteoblastoma
Source: J Pathol. 2025 Dec 22;268(3):263–75. doi: 10.1002/path.70010 (PMC12908201; doi:10.1002/path.70010)
Supplement: Supplementary file 1 — Figure S1. Histology and FOS immunohistochemistry of fMSCs Figure S2. Heatmap for genes relevant in prostaglandin synthesis pathway, in which clear upregulation of different genes is present in FOSΔ compared to pLV Table S1. Primers [file PATH-268-263-s006.docx]

**Truncated FOS impairs osteogenic differentiation and induces prostaglandin and NFκB signalling in an *in vitro* cell-of-origin model for osteoid osteoma and osteoblastoma**

SW Lam *et al. J Pathol* <https://doi.org/10.1002/path.70010>

**Supplementary Figures S1 and S2**

**Supplementary Table S1**

**Supplementary Table S2 is provided as a separate Excel file**

**Supplementary Data S1–S4 are provided as separate Excel files**


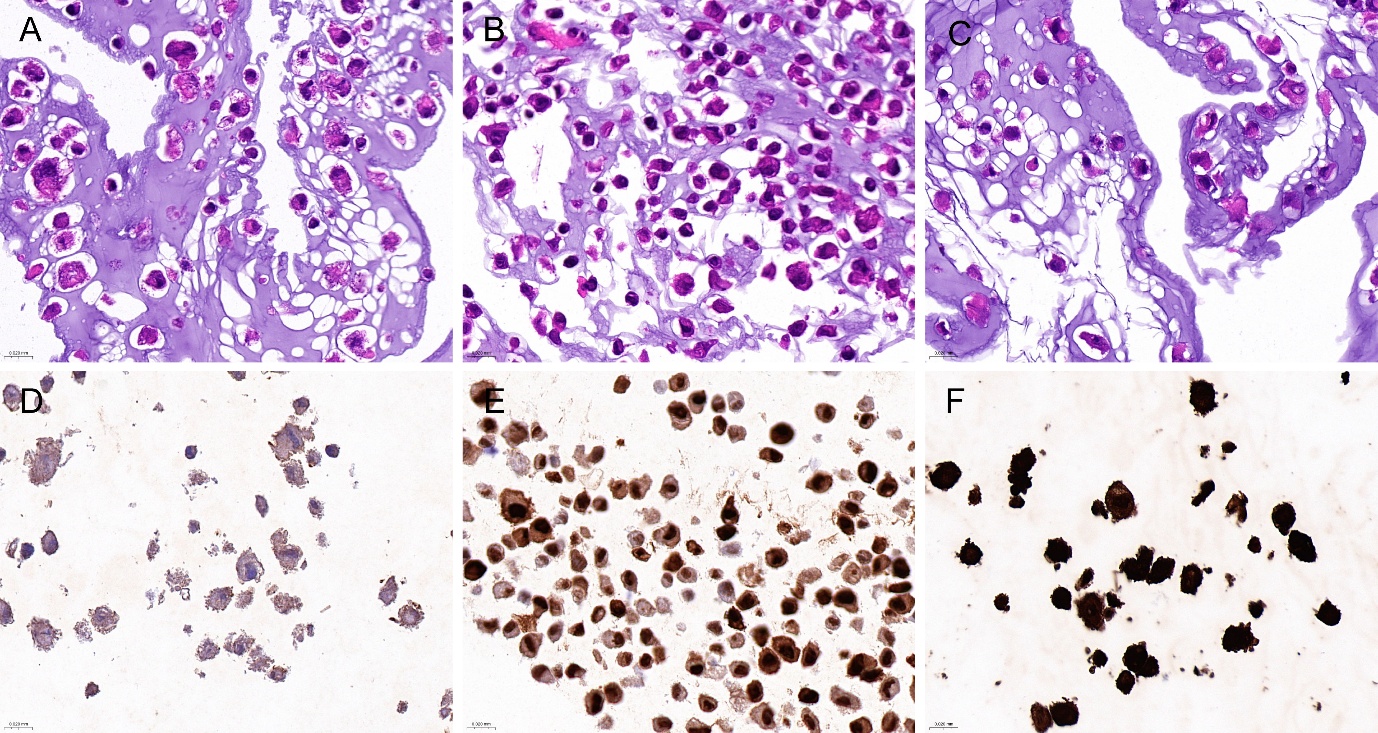


**Figure S1**. **Histology and FOS immunohistochemistry of fMSCs.** (A–C) H&E staining of (A) control fMSCs transduced with pLV, (B) fMSCs transduced with FOS FL, and (C) fMSCs transduced with FOSΔ. (D–F) FOS immunohistochemistry showing (D) absence of expression in pLV, while strong nuclear expression was observed in (E) FOS FL and (F) FOSΔ.


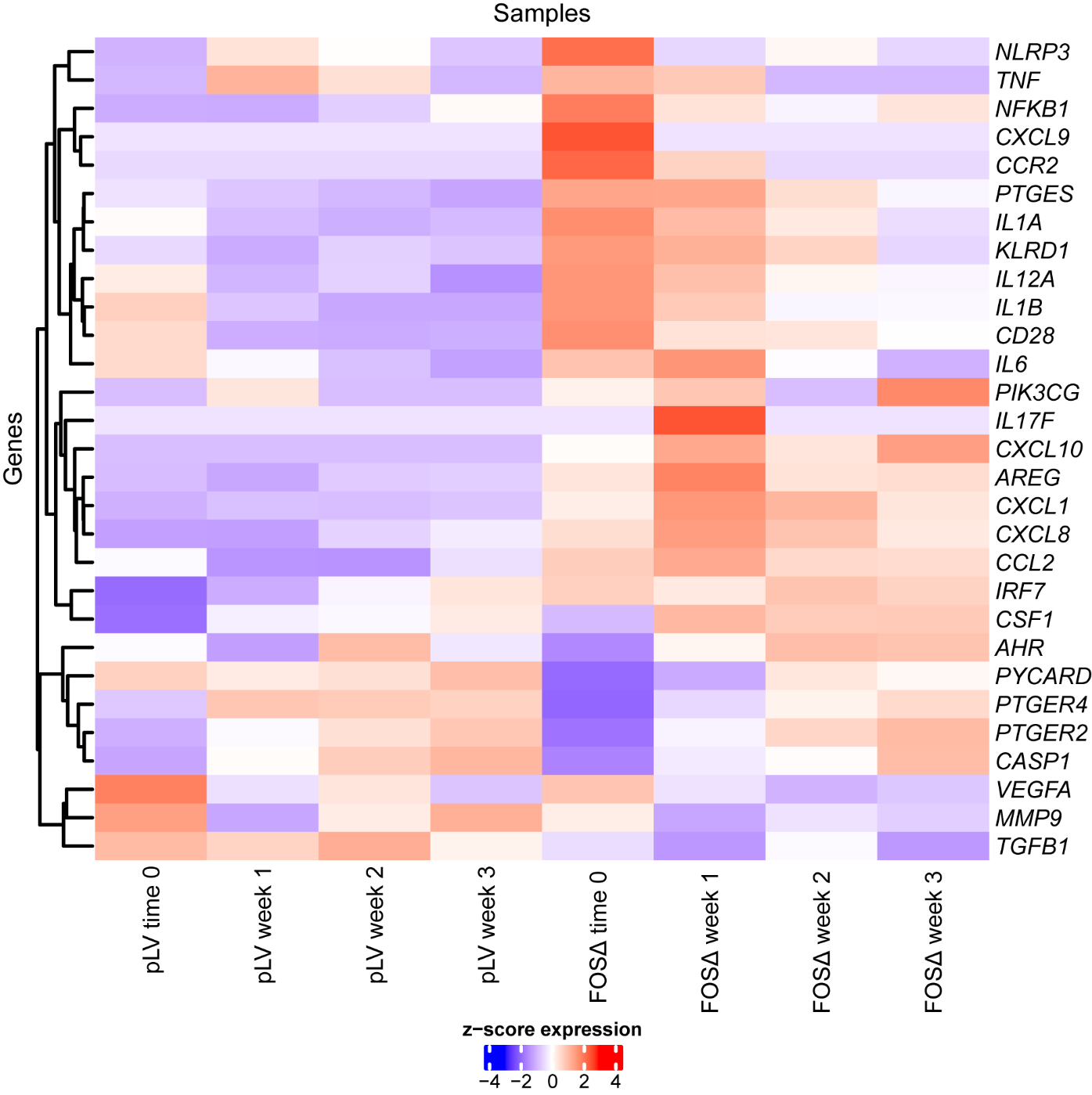


**Figure S2**. Heatmap for genes relevant in prostaglandin synthesis pathway, in which clear upregulation of different genes is present in FOSΔ compared to empty lentiviral vector (pLV). Most upregulated genes in FOSΔ involve different immune regulatory members of the CXC chemokine family, the interleukin gene family, and prostaglandin synthesis (PTGES). VEGFA and TFGB1, genes involved in angiogenesis and the regulation and proliferation of cells, as well as mMP9, involved in extracellular matrix degradation, are downregulated in FOSΔ.

**Table S1.** Primers.

| **Gene** | **Forward primer** | **Reverse primer** |
| --- | --- | --- |
| *ALPL* | TCACTCTCCGAGATGGTGGT | GCCTGCTTGGCTTTTCCTTC |
| *FOS* | GAGAAAAGGAGAATCCGAAGG | GTCAGAGGAAGGCTCATTGC |
| *GAPDH* | TTCCAGGAGCGAGATCCCT | CACCCATGACGAACATGGG |
